# *Mycobacterium tuberculosis* Proteasome Accessory Factor A (PafA) Can Transfer Prokaryotic Ubiquitin-Like Protein (Pup) between Substrates

**DOI:** 10.1128/mBio.00122-17

**Published:** 2017-02-21

**Authors:** Susan Zhang, Kristin E. Burns-Huang, Guido V. Janssen, Huilin Li, Huib Ovaa, Lizbeth Hedstrom, K. Heran Darwin

**Affiliations:** a Department of Microbiology, New York University School of Medicine, New York, New York, USA; b Department of Chemical Immunology, Leiden University Medical Center, Leiden, The Netherlands; c Van Andel Research Institute, Grand Rapids, Michigan, USA; d Departments of Biology and Chemistry, Brandeis University, Waltham, Massachusetts, USA; Harvard School of Public Health

## Abstract

The protein degradation machinery of *Mycobacterium tuberculosis* includes a proteasome and a ubiquitin-like protein (Pup). Proteasome accessory factor A (PafA) attaches Pup to proteins to target them for degradation by the proteasome. Free Pup is unstable and never observed in extracts of *M. tuberculosis*, an observation that led us to hypothesize that PafA may need alternative sources of Pup. Here, we show that PafA can move Pup from one proteasome substrate, inositol 1-phosphate synthetase (Ino1), to two different proteins, malonyl coenzyme A (CoA)-acyl carrier protein transacylase (FabD) and lonely guy (Log). This apparent “transpupylation” reaction required a previously unrecognized depupylase activity in PafA, and, surprisingly, this depupylase activity was much more efficient than the activity of the dedicated depupylase Dop (deamidase of Pup). Thus, PafA can potentially use both newly synthesized Pup and recycled Pup to doom proteins for degradation.

## IMPORTANCE

Unlike eukaryotes, which contain hundreds of ubiquitin ligases, Pup-containing bacteria appear to have a single ligase to pupylate dozens if not hundreds of different proteins. The observation that PafA can depupylate and transpupylate *in vitro* offers new insight into how protein stability is regulated in proteasome-bearing bacteria. Importantly, PafA and the dedicated depupylase Dop are each required for the full virulence of *Mycobacterium tuberculosis*. Thus, inhibition of both enzymes may be extremely attractive for the development of therapeutics against tuberculosis.

## INTRODUCTION

Proteasomes are found in all domains of life and function to degrade proteins in a regulated manner (1). Both in prokaryotes and in eukaryotes, a small protein is used to posttranslationally mark proteins for destruction by a proteasome. However, while the proteasome core proteases are highly similar among the domains of life, the structures of the posttranslational modifications, as well as the mechanisms of their attachment to doomed proteins, have little in common. In eukaryotes, doomed proteins are modified with ubiquitin (Ub), a highly structured and very stable protein with a characteristic β-grasp fold. In contrast, bacteria use an intrinsically disordered protein, Pup, to mark proteins for destruction. In addition to these structural differences, the enzymology of pupylation is unlike that of ubiquitylation. In general terms, Ub is attached to a lysine on a target protein via a cascade of three enzymes: E1, E2, and E3. The carboxyl (C) terminus of Ub has a glycine that is activated by adenylation with ATP and then transferred to an Ub conjugating enzyme (E2), where the Ub glycine forms a thioester bond with a cysteine in the E2. From the E2, Ub can be transferred to various types of Ub ligases or E3s, which finally attach Ub to a lysine in a target protein (reviewed in reference 2).

**FIG 1:**
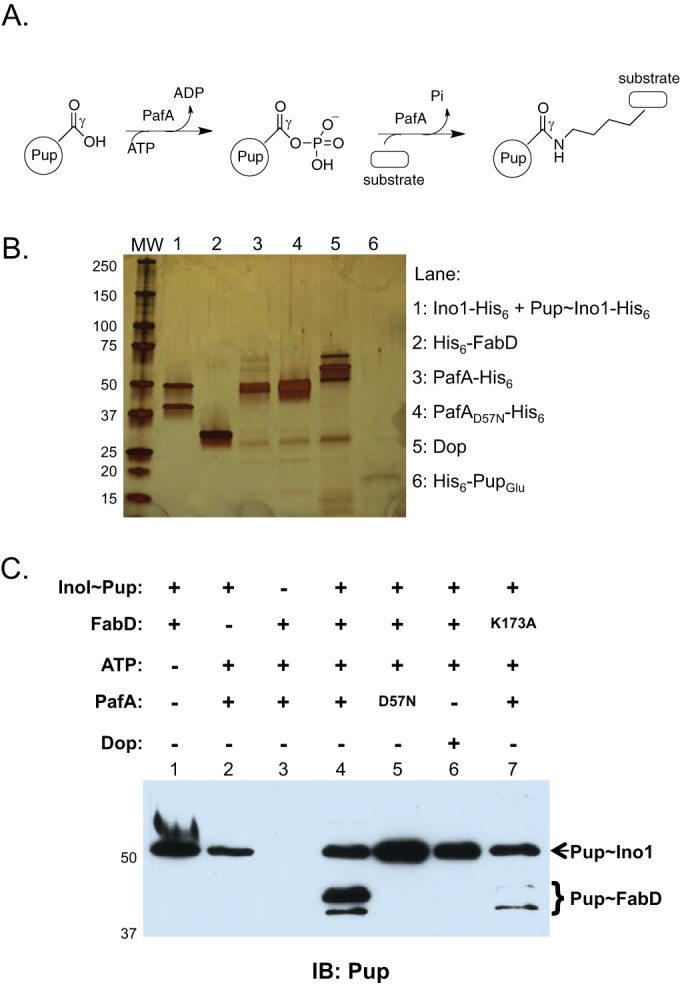
PafA catalyzes transpupylation. (A) Pupylation by PafA. PafA uses ATP to phosphorylate Pup_Glu_ at the γ-carboxylate position, which can then be attacked by the ε-amino group of a lysine on a protein substrate, resulting in the formation of an isopeptide bond between glutamate and lysine. (B) Pupylation enzymes and substrates were purified from either *E. coli* or *M. smegmatis* (Δ*dop*::*aph* mutant) as described in Materials and Methods. Protein was loaded onto a 4 to 15% TGX gradient gel (Bio-Rad) and silver stained. Note that Ino1 forms tetramers in which two molecules in each tetramer are pupylated; therefore, Ino and Pup~Ino1 purify as a 2:2 complex (9). (C) Transfer of Pup from Pup~Ino1 to FabD mediated by PafA requires ATP. Assays were set up as described in Materials and Methods. Reaction mixtures were incubated for 5 h. Molecular weight markers (MW) are shown to the left of each blot or gel. IB, antigen recognized by antibodies used for immunoblotting. For Pup immunoblots, monoclonal antibodies to* M. tuberculosis* Pup were used.

In contrast, Pup is attached to target proteins via the gamma carboxylate (γ-carboxylate) of the C-terminal glutamate residue. In some species, such as *Mycobacterium tuberculosis*, the C-terminal amino acid is glutamine (Pup_Gln_), and the enzyme Dop (deamidase of Pup) must convert this glutamine to glutamate (3). This step is required in order to allow the Pup ligase, PafA, to use ATP to phosphorylate (in contrast to adenylate for Ub) the γ-carboxylate of the terminal glutamate. The ε-amino group of a lysine in a doomed protein can then attack the phosphoglutamate to form an isopeptide bond (4) (Fig. 1A). This reaction is similar to glutamine synthesis, which is a condensation reaction between glutamate and ammonia. Along these lines, PafA is a member of the glutamine synthetase (GS) family of proteins (3, 5, 6). Interestingly, Dop also has structural similarity to PafA and other GS family proteins, although its function is distinct (7). Importantly, in addition to deamidating Pup_Gln_ to Pup_Glu_, Dop can remove Pup from proteins, which can rescue them from proteasomal degradation (6, 8, 9).

There were two observations that led us to speculate that pupylated proteins might be able to donate Pup for new pupylation reactions: first, free Pup is rarely observed in total cell lysates of *M. tuberculosis* unless it is overproduced (6, 10); second, some proteins that are established targets of pupylation do not appear to be rapidly degraded (11). Taking these observations together, we hypothesized that pupylated proteins themselves represent a potentially available pool of Pup for PafA. In this work, we tested the hypothesis that Pup could be transferred between two different proteins and found that PafA could indeed transfer Pup from one proteasome substrate to another. We found that PafA first removed Pup from one substrate in order to then pupylate a new protein. We found that PafA, unlike Dop, could not deamidate Pup_Gln_ to Pup_Glu_; thus, PafA amidase activity appears to be limited to pupylated proteins. Collectively, our data indicate that, in addition to the deamidase and depupylase activities of Dop, *M. tuberculosis* PafA has a depupylase activity that functions to transfer Pup between substrates or depupylate certain proteins under specific conditions.

## RESULTS

### PafA can transfer Pup between two proteasome substrates.

We purified *M. tuberculosis* Myc-Pup~His_6_-FabD (where “~” indicates an isopeptide bond between Pup and the indicated protein) and PafA-His_6_ from *Escherichia coli*, which does not encode a Pup-proteasome system and does not have depupylase or Pup ligase activity (12, 13). Pupylated Ino1 (Pup~Ino1-His_6_) was purified from a *Mycobacterium smegmatis* strain with a deletion and disruption in *dop*, encoding the only known deamidase/depupylase in mycobacteria (14) (kind gift from E. Weber-Ban). We used untagged *M. smegmatis* Dop copurified with *M. tuberculosis* Pup lacking the first 30 amino acids and with a His_6_ epitope tag (His_6_-Pup91); coproduction of His_6_-Pup91 allowed the production of soluble and active Dop. All components were isolated at high purity (see Materials and Methods) as assessed by silver staining of an SDS-PAGE gel with each of the key components (Fig. 1B). Importantly, no free Pup was detected in any of the samples except in the His_6_-Pup_Glu_ lane (Fig. 1B, last lane).

We next tested if Pup could be transferred from Pup~Ino1 to FabD. We started with Pup~Ino1 because it was easy to purify large amounts of protein. Incubation of the “donor” (Pup~Ino1) with the “recipient” (FabD) and PafA showed the emergence of pupylated FabD and a reduction in the amount of Pup~Ino1 (Fig. 1C, lane 4). Reactions using *M. tuberculosis* PafA with aspartate 57 (Asp57) mutated to asparagine (PafA_D57N_), which is required for pupylation (6), did not result in Pup transfer (Fig. 1C, lane 5). No Pup transfer was observed in the presence of Dop (Fig. 1C, lane 6).

FabD is pupylated on a preferred lysine (Lys173 in *M. tuberculosis* FabD), and mutagenesis of Lys173 to alanine results in the stabilization of FabD in *M. smegmatis* (10). Likewise, we found that the mutagenesis of FabD Lys173 to alanine also resulted in reduced transfer of Pup from Pup~Ino1 (Fig. 1C, lane 7). As observed previously, other lysines could still be pupylated in the absence of the preferred Lys173 (12).

**FIG 2:**
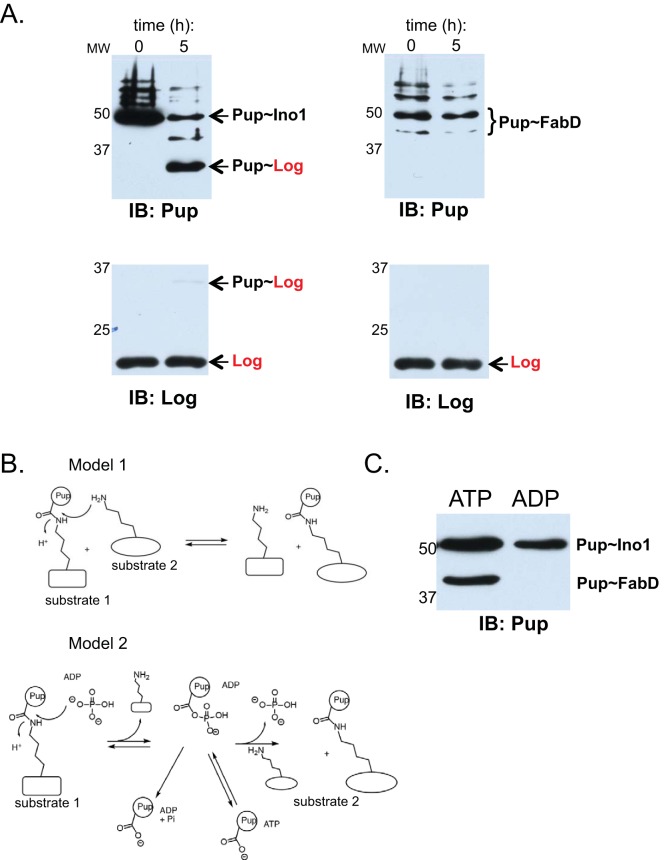
Pup~Ino1 is a better Pup donor than Pup~FabD, and transpupylation requires ATP. Immunoblots of transpupylation reactions performed using purified components shown in Fig. 1 are shown. (A) PafA transfers Pup from Ino1 to Log (left panels) and could not transfer Pup from FabD to Log (right panels). Molecular weight markers (MW) are shown to the left of each blot. (B) Potential models of ATP-independent transpupylation. (C) PafA requires ATP to transfer Pup from Ino1 to FabD. IB, antigen recognized by antibodies used for immunoblotting.

We next tested if Pup~Ino1 could donate Pup to another established proteasome substrate, Log (lonely guy) (15). As with FabD, we saw transfer of Pup from Ino1 to Log (Fig. 2A, left). We then wondered if another pupylated protein could donate Pup to Log. We previously reported a method to purify pupylated FabD (Myc-Pup~FabD-His_6_) from *M. smegmatis*. In contrast to what we observed for transfer from Pup~Ino1 to Log, Pup was not transferred from Pup~FabD to Log under the conditions tested (Fig. 2A, right).

### PafA needs depupylase activity to transfer Pup between proteins.

Pupylation is a two-step reaction in which PafA uses ATP to phosphorylate a carboxylate on the C terminus of Pup, which is then attacked by the amino group of a lysine side chain (4) (Fig. 1A). If PafA were to transfer Pup from one protein to another, a model would be that PafA facilitates the attack of an isopeptide bond in a pupylated protein (“substrate 1”) by an amino group of a lysine of another substrate (“substrate 2”), and that ATP hydrolysis would not be needed; this would resemble transglutamination (Fig. 2B; Model 1). Another possibility is that the presence of free phosphate (in our reaction buffer) in the active site could push a reverse reaction in which phosphate attacks the isopeptide bond between Pup and a donor protein, resulting in the reformation of a Pup-acylphosphate intermediate that could then be attacked by the amino group of a lysine in another, recipient protein (Fig. 2B; Model 2). If either model were possible, the reaction should proceed in the absence of ATP hydrolysis. To test this hypothesis, we performed the transpupylation reaction in the presence of either ATP or ADP. We found that only in the presence of ATP could PafA catalyze the transfer of Pup from Ino1 to FabD (Fig. 2C).

**FIG 3:**
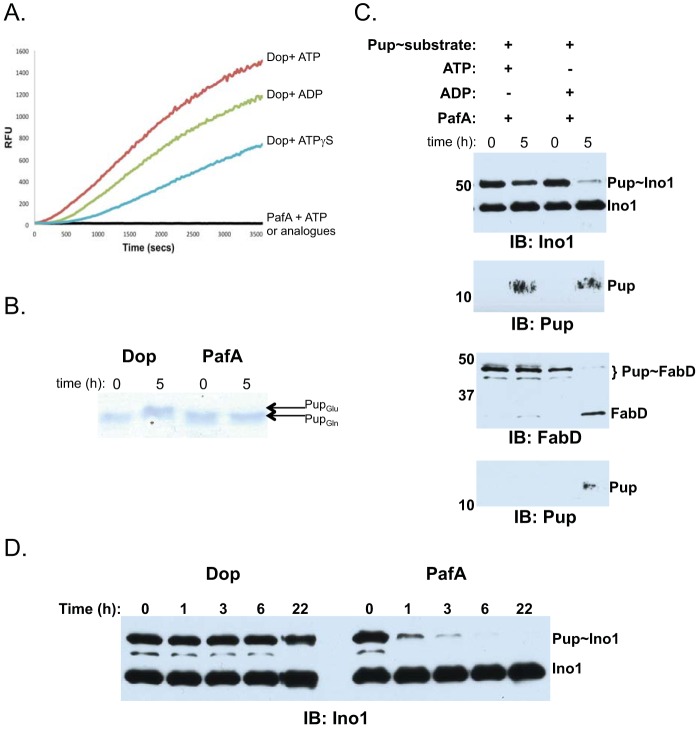
Transpupylation requires depupylase activity by PafA. (A) PafA cannot liberate AMC from Pup30Δ_Q_-AMC in ATP, ADP, or ATPγS. (All data are combined into the black line on the *x* axis). The orange, green, and blue lines represent data determined using purified Dop as a control. RFU, relative fluorescence units. (B) PafA cannot deamidate Pup_Gln_ to Pup_Glu_. Pup_Gln_ (5 μM) was incubated with 0.5 µM Dop or PafA with 5 mM ADP in phosphate buffer. Proteins were separated by 15% SDS-PAGE and stained with Coomassie brilliant blue. (C) PafA can depupylate Pup~Ino1 and Pup~FabD. (D) PafA is a faster depupylase than Dop. Equimolar amounts of either Dop or PafA (0.5 µM) were incubated with 0.25 µM Pup~Ino1 and ADP for 5 h using the buffer described in Materials and Methods. Proteins were separated on a 10% SDS-PAGE gel and detected by immunoblotting with antibodies to Ino1.

On the basis of this result, we hypothesized that PafA must first free Pup_Glu_ from a substrate and phosphorylate the C terminus of Pup_Glu_ (per the known activity of PafA in pupylation) in order to transfer it to a new target. However, PafA has not previously demonstrated depupylase activity; the only characterized depupylase is Dop. There are three established assays for measuring depupylase activity. The first assay that we tested is based on a highly specific activity-based probe for Dop, Pup30Δ_Q_-aminomethylcoumarin (Pup30Δ_Q_-AMC); upon incubation with Pup30Δ_Q_-AMC, Dop rapidly releases fluorescence, and this activity is specific to Dop since lysates made from a *dop M. tuberculosis* mutant, which still have PafA, do not result in the emission of fluorescence (13). Consistent with this previous result, purified PafA could not release AMC from Pup30Δ_Q_-AMC under any condition that we tested (Fig. 3A).

The next assay that we used directly tested whether or not PafA can deamidate the C terminus of Pup_Gln_ and convert it to Pup_Glu_ (3). This simple assay is based on the altered migration of Pup through an SDS-PAGE gel. Consistent with the Pup30Δ_Q_-AMC result, PafA, unlike Dop, could not deamidate Pup_Gln_ to Pup_Glu_ (Fig. 3B).

Although PafA could not hydrolyze Pup30Δ_Q_-AMC or deamidate Pup_Gln_, we could not rule out the possibility that PafA had amidase activity specific to that of a pupylated protein. In addition, the reason that it was previously difficult to observe depupylation by PafA might have been that the presence of ATP would allow PafA to religate Pup to proteins. Therefore, we hypothesized that using ADP instead of ATP would prevent repupylation of a Pup donor protein. To test this hypothesis, we incubated PafA with Pup~Ino1 or Pup~FabD with ADP or ATP and in the absence of a recipient protein. We observed robust depupylation of both Pup~Ino1 and Pup~FabD with ADP (Fig. 3C). Depupylation appeared less efficient with ATP; it is likely that the presence of ATP resulted in the repupylation of the donor proteins. Finally, we wondered how the depupylase activity of PafA compared to that of Dop. Remarkably, PafA was able to depupylate Pup~Ino1 to completion in nearly 1 h whereas Dop could not completely depupylate Pup~Ino1 even after an overnight incubation under these conditions (Fig. 3D).

## DISCUSSION

**FIG 4:**
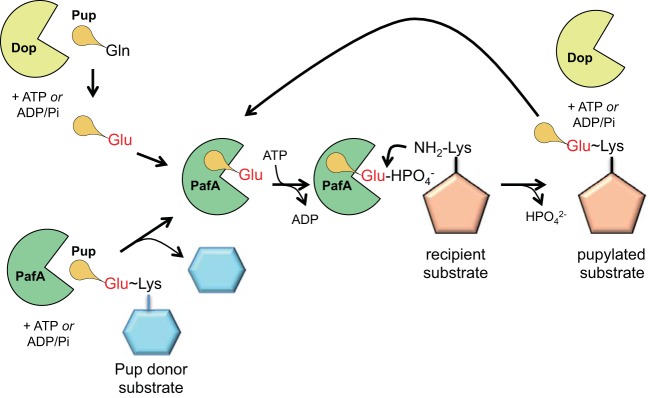
Overview of pathways to pupylation. In the established pupylation pathway, Dop first deamidates *de novo*-synthesized Pup_Gln_ to Pup_Glu_. PafA then phosphorylates Pup_Glu_ at its C terminus, which allows it to be attacked by the amino group of a lysine on a target protein (top). In the present work, we show that PafA can also remove Pup_Glu_ from a protein and ligate it to another substrate (bottom). This process likely requires phosphorylation of Pup_Glu_ as is observed in the established pupylation pathway. Dop can also remove Pup to recycle it for new pupylation reactions by PafA.

In this work, we tested the hypothesis that the only known Pup ligase could catalyze the transfer of Pup from one protein to another. We found that PafA could indeed move Pup from the proteasome substrate Ino1 to FabD or Log, each of which is also an established proteasome substrate (15, 16). However, rather than directly transferring Pup from one protein to another, we found that PafA removes Pup from a donor substrate and then likely catalyzes a “standard” pupylation reaction (Fig. 1A and 4). Strikingly, we also found that the activity of PafA was more robust than that of Dop as a depupylase of Pup~Ino1 under the conditions tested (Fig. 3D). It remains to be determined if PafA has robust depupylase activity with all pupylated proteins or with just a subset of substrates. We also found that Ino1 appeared to be a better Pup donor than FabD, although this might be because FabD is a better recipient that gets repupylated rather than donating Pup to another substrate. Importantly, unlike Dop, PafA could not deamidate Pup_Gln_ to Pup_Glu_ *in vitro* (Fig. 3B), supporting previous observations showing that, even in the presence of PafA, Dop is required to deamidate Pup_Gln_ *in vivo* (6, 14).

We previously showed that aspartate 95 (Asp95) in *M. tuberculosis* Dop could possibly be a direct nucleophile to attack the carbonyl of Pup in an isopeptide bond (17). While our proposed model of Asp95 directly attacking an amide bond remains to be definitively established, we know that it is absolutely required for activity (6). Interestingly, *M. tuberculosis* PafA has a conserved aspartate at an equivalent position, Asp57, based on the comparison of crystal structures, and is also required for pupylation in *M. tuberculosis* (6, 7). In this study, we also showed that Asp57 is required for transpupylation (Fig. 1C, lane 5). Thus, we propose that PafA and Dop use a shared mechanism for depupylation. While it might seem contradictory for Asp57 to participate in both ligase and depupylase activities, it is possible that Asp57 serves two functions: one to properly orient ATP to phosphorylate Pup_Glu_ and the other to act as a direct nucleophile for depupylation. Alternatively, Asp57 may serve to coordinate water or phosphate to hydrolyze an amide bond.

The transfer of Pup from Ino1 to a recipient (FabD or Log) appeared more efficient than transfer from FabD to a recipient. This observation may be simply a consequence of the fact that smaller proteins are better Pup recipients. For example, FabD is a 28-kDa monomeric protein, in contrast to Ino1, which forms tetramers of 140 kDa; thus, it is highly likely that FabD or Log (40-kDa dimer) has better access to the active site of PafA for pupylation.

We previously showed that the ectopic expression of *pup*_*Glu*_ in an *M. tuberculosis dop* mutant does not restore the pupylome; this is most likely because Pup cannot be rescued from proteasomal degradation and recycled in the absence of Dop (6). Based on this previous observation, our present data suggest that PafA either does not recycle Pup or cannot efficiently rescue enough Pup from pupylated proteins to maintain the pupylome. Thus, Dop appears to be continuously needed to deamidate *de novo*-synthesized Pup_Gln_ in order to maintain pupylation *in vivo*.

It remains to be determined if PafA depupylates *in vivo*, an activity that will be difficult to test. One might predict that the mutagenesis of pupylated lysines of Pup donors could affect the stability of nearby proteins that are targets for (trans)pupylation. It is also intriguing to speculate that there might be localized differences in ATP/ADP levels within a cell which might affect the activity of PafA. Under conditions of nutrient starvation when ATP could be limiting, PafA may function more as a depupylase to prevent targeting proteins for degradation. Nothing is known about how PafA selects proteins for pupylation or how pupylated proteins are guided to the proteasome; thus, knowledge of the presence of a robust depupylase activity in PafA may begin to help us understand why certain substrates are more likely to be degraded than others.

## MATERIALS AND METHODS

### Bacterial strains, growth conditions, plasmids, and primers.

See Table 1 for a list of the bacterial strains, plasmids, and primers used in this work. *E. coli* cultures were grown in Luria-Bertani (LB) broth (Difco) or on LB agar at 37°C. For *E. coli*, antibiotics were added with final concentrations of 100 µg ml^−1^ kanamycin or 150 µg ml^−1^ hygromycin. For *M. smegmatis*, we grew bacteria in 7H9 broth (Difco) supplemented with 0.2% glycerol and 0.05% Tween 80 supplemented with 50 µg ml^−1^ hygromycin.

**TABLE 1:** Bacterial strains used in this work

Strain	Relevant genotype or description^a^	Source or reference
*E. coli* ER2566	F^−^ λ^−^ *fhuA2* (*lon*) *ompT lacZ*::*T7 geneI gal sulA11* Δ(*mcrC-mrr*)*114*::IS*10 R*(*mcr-73*::mini-Tn*10*)*2 R*(*zgb-210*::Tn*10*)*1* (Tet^s^) *endA1* (*dcm*)	20
*E. coli* EHD533	ER2566 with pET24b(+)-*his_6_-pup_Gln_*	10
*E. coli* EHD622	Kan^r^; ER2566 with pET24b(+)-*his_6_-fabD*	11
*E. coli* EHD1487	Kan^r^; ER2566 with pET24b(+)-*Log-his*_*6*_	15
*E. coli* EHD1491	Amp^r^ Kan^r^; ER2566 with pDUET-*his_6_-pup91-Msmdop*, pGroESL	This work
*E. coli* EHD1543	BL21(DE3) with pTrc-*pafA-his*_*6*_ + pGro7	This work
*E. coli* EHD2113	ER2566 with pET24b(+)-*his_6_-fabD_K173A_*	This work
*E. coli* EHD2316	BL21(DE3) with pTrc*-his_6_-pafA_D57N_* + pGro7	This work
		
*M. smegmatis* SMR5	Wild type	8
*M. smegmatis* Δ*dop*::*aph*	Kan^r^; Δ*dop*::*aph*	8
*M. smegmatis* MsHD731	Hyg^r^; Δ*dop* with poly(G)-rv*pupE-Msmino1-his*_*6*_	This work
*M. smegmatis* MsHD736	Hyg^r^; Δ*dop* with poly(G)-*myc*-rv*pupE-fabD-his*_*6*_	This work

^a^ Amp, ampicillin; Hyg, hygromycin; Kan, kanamycin; Tet, tetracycline; *Msmdop*, *M. smegmatis dop*; *Msmino1*, *M. smegmatis ino1*.

To make strain EHD1491, *M. smegmatis dop* was cloned into the expression plasmid pETDUET (Novagen, Inc.) at MCS2. We mutated Cys438 to serine to prevent dimerization of Dop. We cloned *M. tuberculosis pup* lacking 91 bp with a hexahistidine tag (His_6_) encoded at the N terminus into MCS1. The final plasmid was used to transform *E. coli* harboring plasmid pGroESL (18).

### Purification of Pup~Ino1-His_6_, Myc-Pup~FabD-His_6_, Log-His_6_, His_6_-FabD, PafA-His_6_, His_6_-Pup_Gln_, and Dop/'Pup-His_6_.

We purified Pup~Ino1-His_6_, Myc-Pup~FabD-His_6_, His_6_-Pup_Gln_, and Log-His_6_ as described elsewhere in detail (9, 10, 15, 19). For PafA-His_6_, a 15-ml starter culture of strain EHD 1543 was grown overnight at 30°C before inoculation into a 1-liter culture of LB media containing 2 mg/ml of arabinose, 50 μg/ml ampicillin, and 30 μg/ml chloramphenicol. Bacteria was grown at 30°C to an optical density at 600 nm (OD_600_) of 0.4 to 0.6 and chilled to 20°C before the addition of isopropyl β-d-1-thiogalactopyranoside (IPTG) to reach a final concentration of 0.6 mM. Cells were further grown overnight (16 to 18 h) with shaking at 20°C before harvest. After resuspension in 50 ml of ice-cold buffer A (50 mM Na_2_PO_4_ [pH 8], 500 mM NaCl, 10% glycerol), cells were lysed by sonication, loaded onto a nickel-nitrilotriacetic acid (Ni-NTA) column, washed with 20 ml buffer A, and further washed with 30 ml buffer A–20 mM imidazole. PafA-His_6_ was eluted with buffer B (50 mM Na_2_PO_4_ [pH 8], 500 mM NaCl, 10% glycerol, 150 mM imidazole) into an equal volume of buffer C (20 mM Tris-HCl [pH 8], 2 M NaCl, 10% glycerol) before being subjected to buffer exchange into 50 mM Tris-HCl (pH 8)–2 M NaCl–1% glycerol (Bio-Rad 10DG columns) and stored at −80°C until use. For Dop/'Pup-His6, strain EHD1491 was grown to an OD_600_ of 0.4 to 0.6 before the addition of IPTG to reach a final concentration of 0.6 mM. Bacteria were growth with shaking at 37°C for 4 to 6 h. Bacteria were harvested and processed essentially as described in the QIAexpressionist manual (Qiagen, Inc.).

All proteins were passed through a Superose 6 10/300 GL size exclusion column and purified using an Äkta purifier (GE Healthcare Life Sciences, Inc.).

### Enzyme assays.

For trans/pupylation assays, reaction mixtures contained 0.5 μM enzyme, 0.25 µM “Pup-donor” substrate, 1 µM “Pup-recipient” substrate, 5 mM ATP, 20 mM MgCl_2_, 1 mM dithiothreitol (DTT), 150 mM NaCl, 10% glycerol, and 50 mM Na_2_PO_4_ (pH 8) at 25°C in a final volume of 100 µl. At the indicated times, samples were withdrawn and added to SDS-denaturing sample buffer to stop the reactions. Dop assays were performed essentially as described previously (9, 13), except that we used sodium phosphate buffer.

### Immunoblotting.

For protein analysis, samples were analyzed by SDS-PAGE, transferred to nitrocellulose membranes, and blocked with 3% bovine serum albumin (BSA) for immunoblot analysis. Detection with horseradish peroxidase was performed using SuperSignal West Pico (Thermo Fisher Scientific). Polyclonal and monoclonal antibodies used in this study are described in detail elsewhere (FabD and Ino1 are described in reference 11; Log in reference 15; Pup in reference 6).
